# Supplementary material for: Are carbon emissions trading and green financial instruments synergistic? -Comprehensive quantitative research based on content analysis
Source: PLoS One. 2024 Mar 7;19(3):e0298601. doi: 10.1371/journal.pone.0298601 (PMC10919601; doi:10.1371/journal.pone.0298601)
Supplement: S1 Data — (ZIP) [file pone.0298601.s001.zip › Data/地级市Z_F工作报告.zip!/╔╜╢1⁄2/╬1⁄2╖╗╩╨/└┤╫╘╣1⁄2╓┌║┼í╛┬φ┐╦╩2╛▌═°í┐_2017.docx]

各位代表:

现在,我代表市人民政府向大会报告工作,请予审议,并请市政协委员和其他列席会议的同志提出意见。

一、过去五年的政府工作二号首长

2012年以来,市政府认真贯彻落实中央和省委、省政府决策部署,在市委正确领导下,紧紧依靠全市人民,拼搏进取、扎实工作,顺利完成市十六届人大历次会议确定的目标任务。

这是经济持续健康发展、综合实力大幅提升的五年。地区生产总值由3541.9亿元增加到5522.7亿元,年均增长9.3%。一般公共预算收入由253.9亿元增加到521.5亿元,翻了一番多。社会消费品零售总额达到2514.9亿元,年均增长12.3%。累计实施投资过亿元项目8260个,完成固定资产投资20041亿元。在全省科学发展综合考核中持续名列前茅。

这是产业转型加快推进、现代产业体系日趋完善的五年。三次产业比例由10.1:55.4:34.5调整为8.6:46.4:45。新增“三品一标”农产品809个,新发展新型农业经营主体2.69万家,创建为国家现代农业示范市。规模以上工业主营业务收入跃上1.3万亿台阶,利润、利税年均增长10.2%和10.8%。高新技术产业产值占比达到33.1%,提升7.6个百分点。建立各类总部基地38处,培育引进企事业单位总部(分支机构)2079家。电商交易额年均增长43.6%,成为国家电子商务示范城市、山东省跨境电商综合试验区。5A级物流企业、5A级旅游景区、国际品牌酒店实现零的突破。2016年旅游消费总额达到677.2亿元。

这是创新创业协调推进、发展动能加快转换的五年。全社会研发投入占GDP比重预计达到2.6%。发明专利授权量、国际专利申请量分别增长3.09倍和3.35倍。盛瑞8AT研发及产业化项目获国家科技进步一等奖。高新区获批建设山东半岛国家自主创新示范区,综合实力居全国高新区第23位。引进培育“千人计划”“万人计划”专家86名,泰山产业领军人才达到34名。新增1个国家重点实验室、2个国家级工程技术研究中心、4个国家产业技术创新战略联盟。建成市级以上科技企业孵化器54家、191万平方米。一批高水平的合作创新平台落户建设。省级产品质量检验中心达到10家。连续被评为全国科技进步先进市,创建为国家知识产权示范城市。建成全省首家实体创业大学。累计新登记各类市场主体49万户,其中企业14.1万户。

这是改革开放不断深入、发展活力显著增强的五年。累计承担78项中央、省改革试点任务。完成农村土地承包经营权、宅基地和农村集体建设用地确权登记颁证工作。齐鲁农村产权交易中心建成运营,新型农村合作金融模式在全省推广。在全国先行实施财政资金金融化管理。在全省率先实现市县两级PPP中心全覆盖。探索推行产业竞争性用地、工业用地弹性年期出让、房屋征收“裁执分离”等一系列改革。进出口总额跃居全省第3位,年均增长6.4%。累计到账外资45.4亿美元。建立境外营销中心113家、海外园区3处,达成国际产能合作项目70个。东亚畜牧交易所正式上线启动,进口澳大利亚活体肉牛指定口岸、进口肉类和冰鲜水产品指定口岸获批建设,鲁辽陆海货滚甩挂运输大通道、“一带一路”潍坊国际多式联运大通道、“鲁新欧·青州号”国际货运班列开通运营。全国首个自贸协定出口实施示范区创建取得重要成果。成为中欧城镇化伙伴关系城市和中美低碳生态试点城市。风筝会、鲁台会影响力进一步提升,成功举办两届中日韩产业博览会。

这是城乡区域统筹发展、新型城镇化水平快速提升的五年。深入实施“海城河”统筹发展战略,全市常住人口城镇化率达到58.15%,年均提高2个百分点。坚持陆海统筹、产城融合,举全市之力建设滨海,现代化海洋经济新区发展格局初步形成。中心城市“四个提升”成效显著。建成“潍V”智慧城市云服务平台,创建为全国首批信息惠民试点市和信息消费试点市。“两河”开发十大工程项目完成投资165亿元。县域经济竞争力进一步增强,特色镇建设快速推进,建成农村新型社区220个。重大基础设施加快建设。潍坊港开通国际集装箱业务,3.5万吨级航道通航,年吞吐量突破3700万吨。潍坊机场开通国际航线,年客运量突破50万人次。争取京沪高铁二线途经潍坊,济青高铁、潍莱高铁开工建设,被确定为全国100个区域性高铁枢纽城市和36个高铁物流枢纽城市之一。潍日高速、济青高速改扩建、潍坊南绕城高速顺利推进。新建改造国省道项目15个。国华寿光电厂建成投用,“特高压入潍”工程进展顺利。引黄入峡、引黄入白等工程建成运营,在连年干旱形势下,保障了用水安全。

这是环保整治全面展开、生态环境显著改善的五年。启动实施“三八六”环保行动和环境治理“十大工程”,PM2.5、PM10、二氧化硫、二氧化氮浓度分别比2013年下降32.8%、23.9%、52.9%、18.6%。新建乡镇污水处理厂55座,市控以上重点河流基本消除劣五类水体。实施县市区空气质量生态补偿、流域生态补偿制度。中心城区和县城实现公共自行车系统全覆盖,年骑行量超过7300万人次,新能源公交车占比超过50%。新增城市绿地2900万平方米,建设人工湿地57处,累计造林67万亩,创建为国家森林城市。

这是民生福祉持续增进、社会保持和谐稳定的五年。民生支出占财政支出比重由75.3%提高到80%,承诺办好的民生实事全部落实到位。城乡居民人均可支配收入分别增长49.3%和54.7%。42.4万农村建档立卡贫困人口实现脱贫,309个省定扶贫重点村摘帽。累计新增城乡就业114.6万人。社会保障水平逐年提升,可量化项目保障标准全部达到或超过省均水平。新开工各类保障房3万套,改造棚户区9.6万套。新建改造农村公路1.2万公里,完成道路安防工程3936公里。规模化集中供水覆盖率达到94.7%,农村全部实现了饮水安全。336个中心村完成电网改造。新改扩建幼儿园1246所,中小学标准化率由40%提高到100%。提前两年基本完成国家确定的农村薄弱学校改造任务,所有县市区通过义务教育发展基本均衡县国家验收。被确定为全国养老服务综合改革试点市,成为社会办医国家联系点。全面两孩政策顺利实施,人均预期寿命达到78.5岁。公共文化场馆全部免费向市民开放。农村“七改”成效显著,实现城乡环卫一体化。创建为全国文明城市。被授予“中国画都”称号。国家食品安全城市创建群众满意度和现场检查验收均取得全省最好成绩。安全生产总体平稳,群众安全感测评居全省前列。连续获评全国双拥模范城、全国法治宣传教育先进城市。外事侨务、民族宗教、妇女儿童、老龄、残疾人、审计、统计、人防、地震、气象、史志、档案、海事等工作实现了新的发展。

这是政府职能加快转变、发展环境持续优化的五年。在全省率先发布“三张清单”,压减63%的市级权力。率先推行“五证合一”商事制度改革。探索实行建设工程项目模块化审批、非制造业建设工程项目审批告知承诺制。市县两级行政许可事项全部实现网上办理,政务服务热线实现整合。综合行政执法体制改革扎实推进。开展降低企业成本“711”专项行动,为企业减负161亿元,化解历史遗留问题7728件。制定出台5个政府规章,配合制定3件实体地方性法规。认真执行市人大及其常委会决议决定,自觉把民主政治协商纳入决策程序,共办理人大代表议案建议和政协委员提案2294件。扎实开展党的群众路线教育实践活动、“三严三实”专题教育、“两学一做”学习教育。深入开展党风廉政建设和反腐败工作,全市纪检监察机关审查违纪案件10445件,给予党纪政纪处分9958人。

2016年,面对复杂的国内外经济形势,市政府主动适应引领经济发展新常态,坚持稳中求进总基调,全面落实新发展理念,统筹做好稳增长、促改革、调结构、惠民生、防风险各项工作,圆满完成了市十六届人大五次会议确定的目标任务。地区生产总值增长8%,一般公共预算收入增长9.1%,固定资产投资增长13.2%,社会消费品零售总额增长10.4%,进出口总额增长6.3%,城乡居民人均可支配收入分别增长8.2%和8.1%,城镇登记失业率控制在2.9%,居民消费价格上涨2.4%,全面完成省政府下达的约束性指标,实现了“十三五”良好开局。

各位代表,五年奋斗,硕果累累。这是以习近平同志为核心的党中央坚强领导的结果,是市委团结带领全市人民开拓创新、埋头苦干的结果,是市人大、市政协和社会各界鼎力支持、共同奋斗的结果。在此,谨代表市人民政府,向全市各族人民,向市人大代表、政协委员,向各界人士、驻潍人民解放军指战员、武警官兵和公安干警致以崇高敬意!向关心支持潍坊发展的老领导、老同志和海内外朋友表示衷心感谢!

我们也清醒地看到,发展中还存在不少矛盾和问题。主要是,经济下行压力依然较大,结构性矛盾仍然突出,行业企业分化现象明显。创新能力不够强,中心城市辐射带动力偏弱,资源环境约束趋紧,民生领域存在短板。维护稳定、防控风险压力较大。部分政府工作人员法治观念、服务意识、担当精神、专业能力不强。我们将高度重视这些问题,采取针对性措施,认真加以解决。

二、今后五年的总体要求和目标任务

刚刚闭幕的中国共产党潍坊市第十二次代表大会,提出了今后五年的奋斗目标,谋划了美好发展蓝图。市政府将紧紧围绕市委的总体部署,全力抓好落实。总的要求是:全面贯彻党的十八大和十八届三中、四中、五中、六中全会精神,深入贯彻落实习近平总书记系列重要讲话和视察山东重要讲话、重要批示精神,认真贯彻党中央治国理政新理念新思想新战略,统筹推进“五位一体”总体布局和协调推进“四个全面”战略布局,紧紧围绕市委“一三四七”目标任务、战略重点和全面从严治党新要求,坚持稳中求进工作总基调,自觉践行新发展理念,坚持以供给侧结构性改革为主线,推动更高质量新发展,实现全面建成小康社会,加快建设富裕文明充满活力的现代化强市。

今后五年,市政府将始终坚持以经济建设为中心,进一步拉长优势、补齐短板,统筹推进经济社会持续健康发展,全面完成“十三五”规划目标任务,促进经济实力和竞争力大幅提升、社会文明水平全面提高、人民生活更加富裕幸福,全力实现建设富裕文明充满活力的现代化强市的总目标。始终坚持准确把握新常态新阶段新方位,抢抓新机遇,应对新挑战,以更高的发展定位和标准要求,促进各方面工作创先争优,确保在全面建成小康社会进程中走在全省全国前列,在贯彻新发展理念、推动转型发展进程中走在全省全国前列,在深化重要领域和关键环节改革进程中走在全省全国前列。始终坚持产业转型升级不动摇,努力构筑现代产业体系,打造国内一流的农业现代化基地、国内重要的高端制造业基地和特色服务业基地,大幅提升产业整体实力和竞争力,促进产业迈向中高端,形成10个以上千亿级产业链和产业集群,规模以上工业主营业务收入突破2万亿,着力打造“产业强市”。始终坚持繁荣发展先进文化,全面实施文明提升工程、文化惠民工程、文化产业培育工程、文化传承发展工程、文化品牌打造工程,促进社会主义核心价值观更加深入人心,城市文明程度位居全国前列,现代公共文化服务体系基本建立,文化产业、文化旅游和文化品牌在国内外的影响力和知名度全面提升,着力打造“文化名市”。始终坚持创新发展,进一步完善社会主义市场经济体制,基本建立开放型经济新体制,基本形成科技创新为引领的全面创新,加快构筑现代城镇体系,在增强发展动力和活力、发展动能接续转换、区域城乡协调发展上实现新突破,大幅提升市场活力、创新创业活力、城乡发展活力,着力打造“活力城市”。始终坚持品质立市,全面实施高端高质高效战略,大力弘扬“工匠精神”,把绿色发展理念融入经济社会发展各方面,着力构筑民生保障体系和现代社会治理体系,全面提高农产品、工业品、服务产品质量,大力提升城乡规划建设管理、生态环境、社会文明、市民生活品质,着力打造“品质城市”。

实现这些目标,必须以新发展理念为指导,崇尚创新、注重协调、倡导绿色、厚植开放、推进共享,努力在各项工作中取得新突破。必须坚持稳中求进工作总基调,统筹做好稳增长、促改革、调结构、惠民生、防风险各项工作,促进经济平稳健康发展和社会和谐稳定。必须深入推进供给侧结构性改革,在供需两端发力,加快培育新动能、发展新经济,不断扩大高质量产品和服务供给。必须坚持创新驱动发展战略,以只争朝夕的紧迫感,加快集聚高端要素、拉长优势产业、发展高端产业,着力以创新引领转型升级。必须坚持改革开放,靠改革增活力,靠开放促发展,靠创新解难题,最大限度释放经济社会发展动力。我们一定要按照市委部署要求,凝神聚力、奋发有为、真抓实干,努力开创政府工作新局面。

三、2017年政府工作安排

综合考虑各方面因素,今年经济社会发展主要预期目标是:地区生产总值增长8%左右,固定资产投资增长11%左右,社会消费品零售总额增长10.5%左右,进出口总额增长5%左右,一般公共预算收入增长8.5%左右,城镇登记失业率控制在3.8%以内,居民消费价格上涨3%左右,城乡居民人均可支配收入分别增长8%左右和8.5%左右。全面完成省下达的约束性指标。

(一)着力完成“三去一降一补”年度任务。“三去一降一补”是深化供给侧结构性改革的重要抓手和重点任务。要分类施策去产能,根据行业、企业情况,主动退出一批、倒逼出清一批、优化整合一批、转移消化一批、改造提升一批,钢铁、水泥、炼油、轮胎4个行业产能利用率稳定在80%以上。稳妥推进企业兼并重组、破产清算,加快“僵尸企业”市场出清。要突出重点去库存,制定促进房地产业转型发展的意见,加快消化商业地产和地下车位库存。提升规划设计、开发建设和物业管理水平,促进房地产业转型升级、健康发展。推进工业产品去库存,全面提升产销率。要多措并举去杠杆,着手实施资本市场“千亿融资计划”,规模企业规范化公司制改制任务完成率达到80%以上,力争新增上市、挂牌企业200家。进一步规范政府举债行为,控制政府性债务过快增长。要坚持不懈降成本,深入落实降低企业成本80条措施,研究制定新的政策措施,进一步降低企业成本,打造有竞争力的营商环境。要加大力度补短板,着力补好服务业占比、重大基础设施建设、教育资源配置、扶贫攻坚等方面的短板。

(二)着力加快产业转型升级。瞄准高端化、智能化、绿色化、服务化方向,积极构建现代产业体系,促进产业迈向中高端,加快建设产业强市。

深入推进农业供给侧结构性改革。实施高效特色农业提升行动和绿色农业计划,加快提升农副产品加工率、附加值,增加绿色优质农产品供给。实施农业品牌引领工程,集中推出一批农业区域公用品牌和产品品牌,重点抓好潍县萝卜、寿光蔬菜、青州花卉等特色农产品品质提升和品牌打造。进一步健全农产品质量安全监管体系,争创国家农产品质量安全市。积极培育新型经营主体、服务主体和新型农民。实施农业龙头企业(合作社、大户)培育工程,加快构建与潍坊农业发展水平相适应的新型农业生产经营体系和社会化服务体系。鼓励扶持新产业新业态,拓展农业产业链价值链,加快培育“新六产”。年内新建改建多功能现代农业示范园区50处。加快海洋牧场项目建设。深入实施“藏粮于地、藏粮于技”战略,严守耕地红线,建设粮食高产创建田270万亩,确保粮食生产稳定。做好防汛抗旱工作。

做强优势产业。机械装备、汽车制造、石化盐化、纺织服装、食品加工、造纸包装等产业是我市的优势产业,也是产业转型的重点和关键。要深入落实好6个优势产业年度转型升级计划和重点项目,加快建设传统优势产业转型发展示范区。继续加大技改力度,滚动实施亿元以上技改项目500项,技改投资增长9%以上。加快工业化与信息化深度融合,持续推进智能制造,重点抓好高端柴油机智能工厂、可穿戴智能工厂等20个智能制造示范项目,支持企业申报国家和省两化融合管理体系贯标试点,争创“中国制造2025”示范城市。实施重点产业品牌培育行动,逐行业制定质量品牌提升计划,推动产品档次、质量、品牌一体化升级。支持企业在全球范围内对标、达标、提标,加大品牌创建和国际并购力度,培育一批行业“隐形冠军”。进一步提升潍柴、雷沃、歌尔、盛瑞、晨鸣、豪迈、孚日、希努尔、得利斯等品牌价值和影响力,加快打造“好品潍坊”。优化产业布局,实施聚焦战略,打造特色产业聚集区。鼓励支持龙头企业领办特色产业园区和专业配套园区,加快推进自动变速器、军民融合产业园等园区建设。出台全市化工企业规范发展转型升级意见和化工园区认定管理办法,推动化工产业集聚发展、高端发展、绿色发展,加快建设大型、特大型化工基地。

培育壮大新兴产业。落实好6个重点新兴产业培育方案和年度计划,集中抓好电子信息、生物医药、生物基新材料三个1000亿级和智能装备、节能环保、新能源汽车三个300亿级新兴产业集群培育工作。强化龙头带动,根据企业需求,制定个性化政策,促进资源要素向龙头企业集聚,鼓励支持歌尔、帅克、迈赫、金丝达、大洋泊车等龙头企业做大做强。强化园区承载,着力推进福田互联网汽车、歌尔虚拟现实、生物基新材料、通用航空、测绘地理信息等重点产业园区建设,促进新兴产业集聚发展。强化项目支撑,突出抓好163个重点项目,年内完成投资177亿元。强化基金支持,积极争取国家战略性产业发展基金和先进制造产业投资基金支持,进一步扩大市级新兴产业基金规模。

促进重点服务业提速发展。深入落实9个重点服务业转型升级方案和年度计划,促进生产性服务业与生活性服务业共同繁荣,传统服务业升级与新兴服务业培育齐头并进,年内服务业增加值增长9%左右。打造区域性总部基地,加快建设潍坊总部基地、食品谷总部基地、港口物流基地及远成·潍坊国际智慧物流城、海王医药等总部基地重点项目,年内新引进企事业单位总部(分支机构)100家以上。大力发展现代物流业,落实全市物流业发展规划,加快建设6个专业化物流园区和16个特色物流园,着力打造东北亚重要物流枢纽。繁荣文化创意产业,积极推进文化消费,在民间艺术品、书画、文创等领域加快培育一批文化骨干企业,建设一批特色文化小镇,促进文化与科技、金融、旅游、教育等深度融合,加快建设创意设计中心。整合提升旅游业,扎实推进旅游业创新发展三年行动计划,加快旅游集散中心建设,促进文化旅游、乡村旅游、工业旅游和生态旅游发展,打造全域旅游品牌。加快发展电子商务,深入实施电子商务跨越发展工程,电商交易额增长25%以上。积极培育物联网、健康养老、运动休闲等新兴服务业,大力发展软件、研发、金融、政府服务等领域的服务外包。

(三)着力推动创新创业。始终把创新作为引领经济社会发展的第一动力。

提升科技创新水平。制定实施科技创新三年行动计划,全社会研发投入占生产总值比重提高到2.65%,科技进步对经济增长的贡献率达到58%。强化企业创新主体地位,大力实施“小升高”科技计划,新认定高新技术企业50家以上,高新技术产业产值占比提高1个百分点。加大招院引校力度,加快建设潍坊市科教创新园,支持有条件的县市区建设科教聚集区,年内引进高等教育机构10家以上。鼓励发展新型研发机构。突出人才引领,深化鸢都产业领军人才工程,实施海内外专家引进计划和国内高层次人才集聚工程。研究制定人才强企若干政策措施和引进顶尖人才“一事一议”实施办法。加强企业家队伍建设,健全完善企业家关爱机制,大力弘扬企业家精神。进一步提升合作创新水平,实施国际合作创新行动计划,鼓励企业在重点国家和地区建立研发机构和孵化基地,落实好“十大科技国际化工程”。完善科技成果转移转化机制,建立技术创新公共信息服务平台,培育一批有影响的科技中介服务品牌。加快推进市科技孵化中心、蓝色智谷等创新型孵化器建设,年内新建和改造提升10家科技孵化器,新增孵化器面积20万平方米。强化平台支撑,设立重大科技创新基金,支持重大科技项目和平台建设。加快建设海洋化工等八大产业技术创新联盟。支持高新区推进山东半岛国家自主创新示范区建设,打造全市高新技术产业引领区。加强知识产权保护,做好国家知识产权强市创建、知识产权区域布局试点工作,加快“两中心一平台”建设。

大力促进产业创新。坚持围绕产业链打造创新链,加快建设农业十大创新公共基地,支持寿光争创国家农业高新技术开发区,全力打造现代农业创新发展中心。建设好工业研究院,加快打造十大产业创新中心。着手组建十大未来产业创新发展中心,抢占创新发展主动权。

深入推进大众创业。制定众创空间发展规划,加快构建以众创空间、众创政策、众创服务、众创制度为重点的众创体系,打造特色创业孵化集聚区。鼓励社会力量投资建设众创空间,新增创客空间5家,创客团队80个,新增市级创业孵化示范基地(园区)20处。完善“鸢都创业证”制度,健全“创业大学+高校+辅导站+实训基地”培训体系。推进“双创”政策进高校、进院所、进企业、进社区,支持大学生、退役军人、转岗职工、进城务工人员等投身创业,鼓励各类专业人才引领创业,激励在外人才回乡创业。

深化智慧城市建设。完善智慧潍坊基础设施,形成集政务应用和社会应用于一体的云计算中心,实现城区主要道路和重点区域免费WiFi全覆盖。打造“潍V”智慧城市应用品牌,构建智慧民生服务体系。以“互联网+政务服务”为抓手,统筹推进智慧政务建设和应用。创新大数据、物联网建设运营模式,争创全省大数据交易中心。

(四)着力深化重点领域改革。坚持问题导向,什么制约发展就改革什么,什么影响效能就突破什么,积极推进重要领域和关键环节改革。深化农业农村改革。扎实推进农业综合改革试验区建设。深化农村土地所有权、承包权和经营权“三权分置”,稳步实施农村集体产权制度改革。探索开展农民住房财产权抵押贷款。继续深化供销社综合改革。依法稳妥推进合村并点。搞好财税体制改革。统筹推进预算管理、税收制度改革和市县两级事权与支出责任划分,加快构建现代财政制度。有序推进基本公共服务均等化,逐步实现基本民生标准、农业转移人口市民化待遇、工资性收入政策、运转经费标准和公共设施建设标准“五个基本统一”。继续扩大PPP模式应用,年内全市集中运作项目30个以上,吸引社会投资300亿元以上。进一步完善招投标办法,规范公共资源交易平台,提高资源配置的透明度、公平性。加大金融改革力度。制定实施新一轮金融创新发展计划,打造金融高地。加大金融对实体经济的支持力度,加快建设产业金融创新中心。鼓励各类企业和银行开拓资本市场,进一步提高直接融资比重。推动地方金融机构加快发展,组建地方法人保险机构,支持多样化、特色化金融机构发展。健全完善政府融资担保体系。深化新型农村合作金融试点改革。提升东亚畜牧交易所、齐鲁农村产权交易中心、潍坊文化产权交易中心等交易平台运营水平。增强同风险赛跑意识,进一步完善金融风险防控化解机制,压实金融风险属地管理责任,提升依法处置化解金融风险的能力,严守不发生系统性、区域性金融风险的底线。加快事业单位改革。落实中央关于加强和改进行业事业单位领导人员管理的“五个办法”。清理规范“僵尸”事业单位,深化公立医院和高校人员控制总量备案管理,抓好公立医院、科研院所法人治理结构建设。统筹推进教育、医疗、社保等社会事业改革。扎实做好公务员职务与职级并行制度试点工作。

(五)着力扩大对外开放。开放是转型发展的必由之路。要进一步强化开放发展理念,充分用好“两个市场、两种资源”。深入落实国际化四年工作计划,突出“六个领域”“六个重点”“三大布局”,大力提升开放发展水平。多措并举稳外贸。实施外贸转型升级专项行动,落实和完善稳定出口的政策措施,实施“千企百展”市场开拓计划。支持发展跨境电商、外贸综合服务、市场采购贸易等新业态。加快线上“单一窗口”和线下“综合支撑”平台建设,着力打造跨境电商综合试验区。积极参与鲁贸名品展示中心建设。推广安丘自贸协定出口实施示范区模式,力争全域整建制创建全国自贸协定出口实施示范市。加大招商引资力度。实施利用外资“十项计划”,调整完善招商引资政策,改进招商体制机制。强化专业招商、以商招商、精准招商,发挥龙头企业在产业链招商中的主体作用,进一步提升招商引资成效。深入实施与世界500强、行业领军企业及知名院所机构合作行动方案,开展“外企服务促进年”活动,落实“服务大使”“专家顾问小组”等制度。有重点地“走出去”。深度参与“一带一路”建设,加快建立海外战略支点城市网络。支持优势企业拓展海外高端工程市场,引导企业向设计咨询、运营服务等高附加值领域拓展,带动装备、技术、产能走出去。大力推进“潍营满欧”“潍青欧”海铁联运大通道建设,提升“鲁新欧·青州号”国际货运班列运营能力。推广潍柴动力海外并购经验。支持有条件的企业建设境外生产装配基地、资源开发基地、种植养殖基地。打造开放合作平台。发挥高新区、滨海区对外对内开放龙头作用。完善综合保税区服务功能,打造全市外向型经济服务区。深化开发区体制机制改革,加快开发区转型升级。提升中日韩产业博览会市场化运作水平。建成涉外部门联合服务“网上大厅”。积极对接引进国际规则和服务体系,加快规划建设国际社区、国际学校、国际医疗机构等配套服务设施。加强与青岛在规划、重大基础设施等方面的合作,推进青潍协同发展。做好援疆、援藏等对口支援工作。

(六)着力提升新型城镇化水平。深入推进以人为核心的新型城镇化,进一步完善市民化“1+N”政策体系,实现外来务工人员市民化9.4万人、就地转移人口市民化3.6万人、城中村城边村原有居民市民化12.3万人。常住人口城镇化率提高2个百分点左右。

深入实施蓝色经济区发展规划。全域统筹,突出滨海,着手编制海岸带规划,抓好大家洼、央子、港口经济区等重点片区建设,大力推进海洋科技大学等27个重大支撑项目建设。突出抓好临港经济、海洋动力、绿色化工、海洋生物医药、滨海旅游等重点产业,打造陆海统筹、产城融合发展示范区,加快建设滨海海洋经济新区。

突出中心引领。实施新一轮提升市区计划,落实好“十大专项行动”。扎实推进242个重点提升项目。加快高铁新片区、站南广场、中央商务区、中国食品谷、医疗健康城等重点片区规划建设,大力推进金融小镇、梦幻小镇、坊茨小镇等特色聚集区建设。扎实开展“七项专项整治”,提升城市网格化、精细化、法治化、智慧化管理水平。

推进“一体两翼”。推动“七城”一体布局、相向发展。拉长县域经济优势,支持县域竞相发展。开展中小城市试点,加快推进省级示范镇和特色小镇建设,集中培育1个国家级特色小镇、5个省级特色小镇。深入推进“两区”同建,集中打造100个“四全一特”示范社区,不断增强社区自治和服务功能。加快实施城镇棚户区改造三年计划,年内改造棚户区8.6万套以上。积极推动镇改街、村改居,转变城中村、城边村和农村新建社区管理模式。深入落实农村“七改”计划,新建改造农村公路1300公里,改造农厕12.8万户,完成农村存量危房改造,50%的村庄达到美丽乡村建设标准。

强化基础支撑。加快潍坊港2个航道、11个泊位及业务码头建设,协调推进疏港铁路、鲁辽陆海货滚甩挂运输大通道建设。加快推进济青高铁、潍莱高铁及枢纽配套工程。潍日高速北段、宝通街东延项目建成通车。潍坊南绕城高速、两条市区外环路开工建设。潍日高速南段、济青高速改扩建工程加快建设。加快推进新机场审批立项,同步启动工程设计和征地拆迁。结合城市重点片区规划建设,优化完善实施中心城区交通组织方案,加快推进城市快速路建设。榆横-潍坊、扎鲁特-青州特高压工程建成投运,完成农村电网改造升级“两年攻坚战”。加快黄水东调、引黄入峡后续工程建设。

(七)着力做好民生保障和社会治理。民生和社会治理工作是社会建设的两大根本任务。今年,民生支出达到535亿元以上。进一步加大精准扶贫、精准脱贫力度,确保剩余28个省定扶贫重点村全部摘帽,1.1万农村建档立卡贫困人口全部脱贫。统筹解决城市贫困问题。做好高校毕业生、就业困难人员和去产能转岗再就业职工等重点群体就业工作,落实农民工职业技能提升、权益保障和公共服务3项“三年行动计划”,新增城镇就业10万人。进一步改善办学条件,均衡配置各类教育资源,全市新改扩建中小学77所、普惠性幼儿园117所。完成破解中小学“大班额”和“全面改薄”任务。深化管办评改革,扩大学校办学自主权,全面落实立德树人根本任务。适应高考综合改革,加快普通高中创新发展。深入推进国家职业教育创新发展试验区建设,加快实施高校特色发展计划和重点建设工程。推进基层医疗机构标准化
